# Curcumin-Loaded Nanoparticles Impair the Pro-Tumor Activity of Acid-Stressed MSC in an In Vitro Model of Osteosarcoma

**DOI:** 10.3390/ijms22115760

**Published:** 2021-05-28

**Authors:** Gemma Di Pompo, Margherita Cortini, Roberto Palomba, Valentina Di Francesco, Elena Bellotti, Paolo Decuzzi, Nicola Baldini, Sofia Avnet

**Affiliations:** 1 Biomedical Sciences and Technologies Lab, IRCCS Istituto Ortopedico Rizzoli, 40136 Bologna, Italy; gemma.dipompo@ior.it (G.D.P.); margherita.cortini@ior.it (M.C.); nicola.baldini@ior.it (N.B.); 2 Laboratory of Nanotechnology for Precision Medicine, Istituto Italiano di Tecnologia, 16163 Genova, Italy; Roberto.Palomba@iit.it (R.P.); Valentina.difrancesco@iit.it (V.D.F.); Elena.Bellotti@iit.it (E.B.); Paolo.Decuzzi@iit.it (P.D.); 3 Department of Biomedical and Neuromotor Sciences, University of Bologna, 40125 Bologna, Italy

**Keywords:** tumor microenvironment, mesenchymal stromal cells, curcumin, osteosarcoma, nanoparticle

## Abstract

In the tumor microenvironment, mesenchymal stromal cells (MSCs) are key modulators of cancer cell behavior in response to several stimuli. Intratumoral acidosis is a metabolic trait of fast-growing tumors that can induce a pro-tumorigenic phenotype in MSCs through the activation of the NF-κB-mediated inflammatory pathway, driving tumor clonogenicity, invasion, and chemoresistance. Recent studies have indicated that curcumin, a natural ingredient extracted from *Curcuma longa*, acts as an NF-κB inhibitor with anti-inflammatory properties. In this work, highly proliferating osteosarcoma cells were used to study the ability of curcumin to reduce the supportive effect of MSCs when stimulated by acidosis. Due to the poor solubility of curcumin in biological fluids, we used spherical polymeric nanoparticles as carriers (SPN-curc) to optimize its uptake by MSCs. We showed that SPN-curc inhibited the release of inflammatory cytokines (IL6 and IL8) by acidity-stimulated MSCs at a higher extent than by free curcumin. SPN-curc treatment was also successful in blocking tumor stemness, migration, and invasion that were driven by the secretome of acid-stressed MSCs. Overall, these data encourage the use of lipid–polymeric nanoparticles encapsulating NF-κB inhibitors such as curcumin to treat cancers whose progression is stimulated by an activated mesenchymal stroma.

## 1. Introduction

The tumor microenvironment (TME) consists of several normal host cells in an extracellular matrix containing various solutes, and provides a milieu that enables cancer survival [1]. Under certain conditions, this heterogeneous and complex ecosystem may promote cancer aggressiveness and migration, and contribute to the failure of current anti-cancer therapies. The tumor-infiltrating stroma primarily includes vascular and endothelial cells, adipocytes, cancer-associated fibroblasts (CAFs), immune cells (e.g., macrophages, lymphocytes, and dendritic cells), and mesenchymal stromal cells (MSCs) [2]. In particular, MSCs have been recognized as a crucial element in cancer progression. MSCs are a heterogeneous class of self-renewing, multipotent progenitor cells that reside in the bone marrow, but can also be found in other tissues [3]. Their physiological functions include a tendency to migrate to sites of injury [4], to support the repair and regeneration of damaged tissues [5], and to modulate the immune response [6]. In the TME, MSCs interact with tumor cells and other reactive elements through several paracrine molecules in a complex crosstalk that facilitates tumor growth, metastasis, and resistance to therapy [7,8]. 

MSCs are also key players in osteosarcoma (OS) progression [9,10]. Although relatively rare, OS is the most common primary bone neoplasm in children and adolescents, and a leading cause of cancer-related death in young people [11,12]. In OS, MSCs establish a metabolic coupling with tumor cells [13], and a paracrine communication mediated by pro-tumorigenic exosomes [14] and cytokines (IL6, IL8, and CCL5) [15,16,17,18,19] that eventually leads to the promotion of the stem-like subsets of OS, resulting in increased clonogenicity, migration, stemness, and chemoresistance [20,21]. The pro-tumorigenic activity of MSCs in OS can be further enhanced and modulated by biochemical and physical stimuli, such as extracellular acidosis resulting from the metabolic shift towards aerobic glycolysis of tumor cells [22]. In MSCs, a low pH acts as a driving force for both physiological regeneration [23] and cancer progression. An acidic pH further drives resident or tumor-recruited MSCs to secrete pro-tumorigenic mediators [24,25]. We have extensively shown that a low pH directly influences OS cell behavior [26,27,28,29,30] and reprograms tumor-associated MSCs into an OS-supporting phenotype. Specifically, a low pH activates the NF-κB inflammatory pathway in MSCs by inducing expression of the RelA proto-oncogene NF-κB subunit (RelA), RelB proto-oncogene NF-κB subunit (RelB), and nuclear factor kappa B subunit 1 (NFKB1), as well as nuclear internalization of the NF-κB complex, which, in turn, activates the transcription of IL6 and IL8. 

On these grounds, pharmacological inhibition of the NF-κB pathway in MSCs is particularly attractive as a therapeutic strategy against OS and other tumors characterized by an acidic microenvironment. Among the wide variety of NF-κB inhibitors, curcumin, a natural polyphenolic compound derived from the rhizomes of *Curcuma longa*, is widely used to treat a variety of inflammatory conditions, including cancer, in part because of its high accessibility, cost-effectiveness, and safety [31]. Curcumin inhibits the phosphorylation and degradation of the nuclear factor of kappa light polypeptide gene enhancer in B-cells inhibitor alpha (IκBα), preventing the subsequent translocation of NF-κB into the cell nucleus [32]. As a result, it prevents the transcription of downstream pro-inflammatory proteins and pro-neoplastic factors, and thus can be considered to be a potential anti-cancer drug with multiple activities [33]. Its effects have been extensively demonstrated in leukemia, multiple myeloma, breast, lung, and gastric carcinoma, and in several sarcomas [31,34,35,36,37,38]. Notably, in OS cell lines, curcumin shows tumor-selective cytotoxicity and pro-apoptotic and anti-invasive effects [39,40,41,42,43]. Most recently, curcumin has been shown to enhance the effects of chemo- and radiotherapy on glioblastoma [44].

Despite the growing interest in the anti-cancer effects of curcumin, its application is currently limited by its poor chemical stability, solubility in water, and inadequate oral bioavailability [45]. To overcome these critical issues, different strategies, such as liposome-based formulations, and emulsion or microsphere preparations of the drug, have been tested [46,47,48]. Encapsulation of curcumin in nanosystems is expected to increase its cellular uptake, extend its half-life, and improve its accumulation at cancer sites [49,50]. We have recently developed spherical polymeric nanoparticles (SPN) encapsulating curcumin (SPN-curc), and demonstrated their ability to effectively diminish the vascular deposition of circulating tumor cells (CTCs) from a highly metastatic breast cancer cell line [51], to prevent tumor growth in combination with conventional chemotherapeutic molecules [52], and to counteract amyloid-β fibrils-induced inflammation [53]. 

Although the effects of curcumin on cancer cells have been extensively described, its action on cancer-associated pro-tumorigenic stromal cells has been less extensively explored [54,55]. In this study, in order to test the therapeutic potential of curcumin against OS, we considered the acidic conditions typical of the TME, and the role played by inflammatory MSCs in cancer progression. We also explored the potential of SPN-curc formulation to enhance the anti-inflammatory properties and stability of curcumin and their consequences on the paracrine influence of MSCs on OS cell stemness and invasiveness.

## 2. Results

### 2.1. Cytotoxicity of Curcumin against Osteosarcoma Cells and Anti-Inflammatory Effects on Normal MSCs

**Figure 1:**
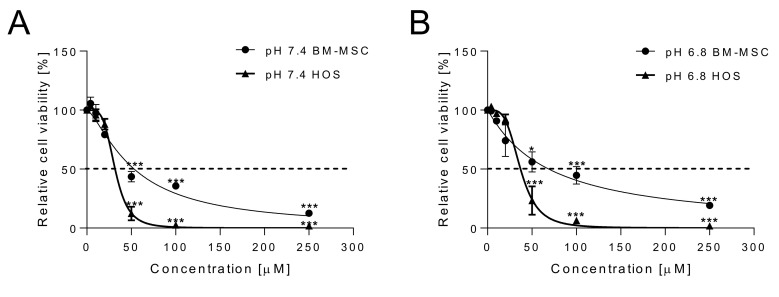
Cytotoxicity of free curcumin in osteosarcoma (HOS) and mesenchymal stromal cells (BM-MSCs). Viability of HOS cells and BM-MSC at 48 h after exposure to increasing concentrations of free curcumin under (**A**) physiological conditions (medium buffered at pH 7.4, *** *p* < 0.001 vs. 0 µM = 100% cell viability) and (**B**) acidic conditions (medium buffered at pH 6.8, *** *p* < 0.001, and * *p* < 0.05 vs. 0 µM = 100% cell viability). Data are expressed as mean ± SE (*n* = 4).

First, we aimed to confirm the selective cytotoxicity of curcumin as an anti-cancer drug under neutral (pH 7.4, Figure 1A) and acidic (pH 6.8, Figure 1B) conditions. We exposed an OS cell line (HOS), in comparison with bone marrow-derived MSCs (BM-MSCs) as control, for 48 h, with increasing drug concentrations, from 4 to 250 μM. Preset acidic pH values were obtained by adjusting the concentration of sodium bicarbonate in the medium. Cell viability was determined by using the acid phosphatase assay. Curcumin appeared to be more cytotoxic to cancer cells than to normal cells under both neutral and acidic conditions. At the neutral pH, IC_50_ values were 31.72 μM and 53.23 μM for HOS and BM-MSCs, respectively. Similar values were also found at the acidic pH, namely, IC_50_ = 37.03 vs. 68.86 μM, revealing a slightly reduced susceptibility of the cells to treatment under acidic conditions. The higher sensitivity to curcumin of cancer cells in respect to normal cells has already been reported as being due to curcumin’s activity against several tumor-specific pathways, including the cell proliferation pathway and the cell survival pathway [39,56,57].

**Figure 2:**
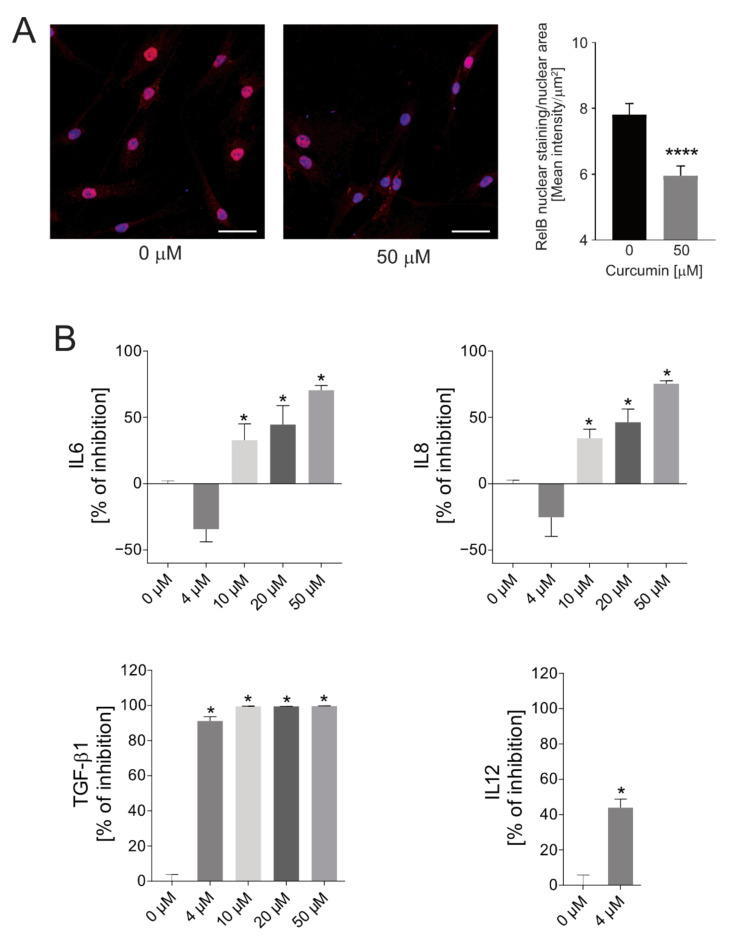
Effects of free curcumin on the NF-κB pathway and the secretion of inflammatory cytokines by acid-treated MSCs. (**A**) RelB immunofluorescence staining of fixed cells that were either pre-treated or not pre-treated with curcumin. Nuclei were counterstained with Hoechst 33258 (scale bar 50 μM). The intensity of RelB staining in the nuclear area was quantified by automatic measurement. Results are shown in the graph in the right panel (**** *p* < 0.0001). (**B**) Inhibition of IL6, IL8, IL12, and TGF-β1 protein secretion by acid-stressed BM-MSCs under acidic conditions (pH 6.8), as assessed by ELISA assay, after 24 h exposure to increasing concentrations of free curcumin. Data are expressed as percentage of curcumin inhibition in respect to the 0 µM concentration. Mean ± SE (*n* = 4, * *p* < 0.05 vs. 0 µM).

Since we previously demonstrated that extracellular acidosis activates the transcriptional inflammatory factor NF-κB complex [21], we then verified whether curcumin effectively impairs the acid-induced secretion of NF-κB-mediated inflammatory cytokines in BM-MSCs. We found a reduced nuclear translocation of RelB in cells treated with curcumin (Figure 2A). RelB is a protein of the NF-κB complex that, as we have previously shown, increases when MSCs are exposed to short-term acidosis, and its activation ultimately leads to enhanced secretion of IL6 and IL8 [21]. Thus, in this model we also assessed the secretion of IL6 and IL8 in response to curcumin treatment via ELISA at increasing concentrations, and by culturing BM-MSCs at pH 6.8. Starting from the 10 µM concentration, free curcumin blocked the acid-induced secretion of both IL6 and IL8 (Figure 2B) in a dose-dependent manner. In contrast, no inhibitory effects were recorded upon BM-MSCs being treated with the lowest concentration of the drug (4 µM); on the contrary, the lowest concentration seemed to cause a paradoxical effect, although this trend was not significant. Finally, as additional inflammatory cytokines that can be affected by curcumin treatment, we also evaluated the concentrations of interleukin 1 beta (IL1β), tumor necrosis factor alpha (TNF-α), transforming growth factor beta 1 (TGF-β1), and interleukin 12 (IL12). Both IL-1β and TNF-α levels were under the detection limit of the ELISA assay (data not shown). Conversely, TGF-β1 secretion was significantly inhibited by curcumin treatment in a dose-dependent manner, and with a strong effect even at the 4 µM concentration (*p* = 0.0209, Figure 2B). The lowest concentration of the drug (4 µM) was also sufficient to reduce IL12 secretion by BM-MSCs (*p* = 0.0339, Figure 2B). For the highest concentrations (10, 20, and 50 μM), IL12 levels were under the detection limit, suggesting a complete inhibition of the protein expression by curcumin treatment.

### 2.2. Curcumin-Loaded SPN (SPN-curc) Fabrication and Characterization

Curcumin-loaded spherical polymeric nanoparticles (SPN-curc) were synthetized using a sonication–emulsion method, as previously described [53,58]. Briefly, an oil phase containing carboxyl-terminated poly(lactic-*co*-glycolic acid) (PLGA), 1,2-dipalmitoyl-sn-glycero-3-phosphocholine (DPPC), and curcumin (curc) was added drop by drop to an aqueous phase containing 1,2-distearoyl-sn-glycero-3-phosphoethanolamine-N-[carboxy(polyethylene glycol)-2000 (DSPE-PEG-COOH) in 4% ethanol, under sonication (100% amplitude for 1.5 min). The so-formed emulsion was magnetically stirred for 3 h to facilitate the organic solvent’s evaporation. Particles were than purified via a series of centrifugation and washing steps, as indicated in the methods section.

**Figure 3:**
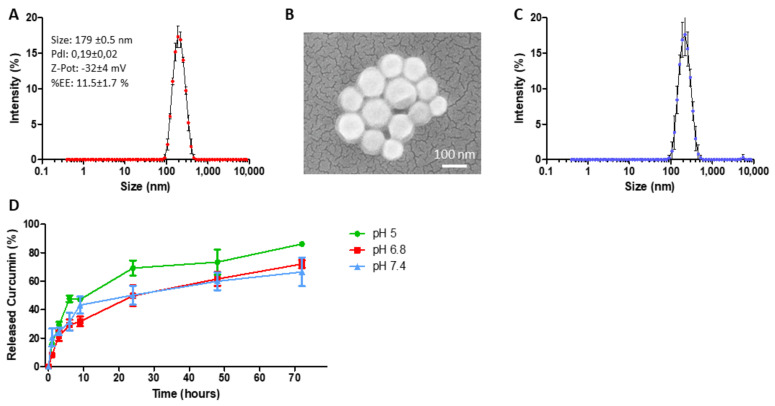
SPN-curc characterization. (**A**) Hydrodynamic diameter of SPN-curc via dynamic light scattering analysis. (**B**) Scanning electron microscopy images of SPN-curc. (**C**) Hydrodynamic diameter of Cy5-SPNs via dynamic light scattering analysis. (**D**) In vitro release profile of curcumin from SPNs at different pH (7.4, 6.8 and 5.0).

The polymeric PLGA core of the nanoparticles, surrounded and stabilized by a phospholipid monolayer, physically entraps the hydrophobic molecules of curcumin. As shown in Figure 3A, SPNs have an average hydrodynamic diameter of 179 ± 0.5 nm, characterized by a polydispersity index (PDI) below 0.2. The surface electrostatic ζ-potential was equal to −32 ± 4 mV; the negative charge arises from the presence of the carboxylate groups of the DSPE-PEG-COOH. It is important to underline that the hydrodynamic size (~200 nm) and the surface properties (negative charge and PEGylation) of the SPNs are ideal for their passive and progressive accumulation within the tumor mass via the enhanced permeability and retention effect (EPR) [59]. The therapeutic molecule curcumin was loaded within the SPNs’ hydrophobic cores, and a spectrophotometer was used to quantify the loading amounts and release profiles. The EE% was measured to be equal to 11.5 ± 1.7%. Figure 3B shows an SEM image of SPN-curc, confirming the homogeneous nanoparticle distribution and its spherical shape. In Figure 3C, the average hydrodynamic diameter, polydispersity index, and surface ζ-potential are provided for the Cy5-labeled SPNs used for the in vitro cell imaging experiments. In Figure 3D, the release profile of curcumin from SPN-curc is reported. The release was determined in a 4 L PBS solution (infinite sink condition) for both the pH 7.4 and pH 6.8 conditions; 4 L of an acetate buffer was used instead for analyzing the release at pH 5. As indicated by the graph, under all of the pH conditions tested, release was fastest in the first 9 h. 24 h later, ~50% of curcumin was released at both pH 7.4 and 6.8, while at pH 5.0 ~70% of curcumin release plateaued. The release profile observed at pH 6.8 was not significantly different with respect to pH 7.4. However, at pH 5, the curcumin release was faster.

### 2.3. SPN-curc Internalization in BM-MSCs

**Figure 4:**
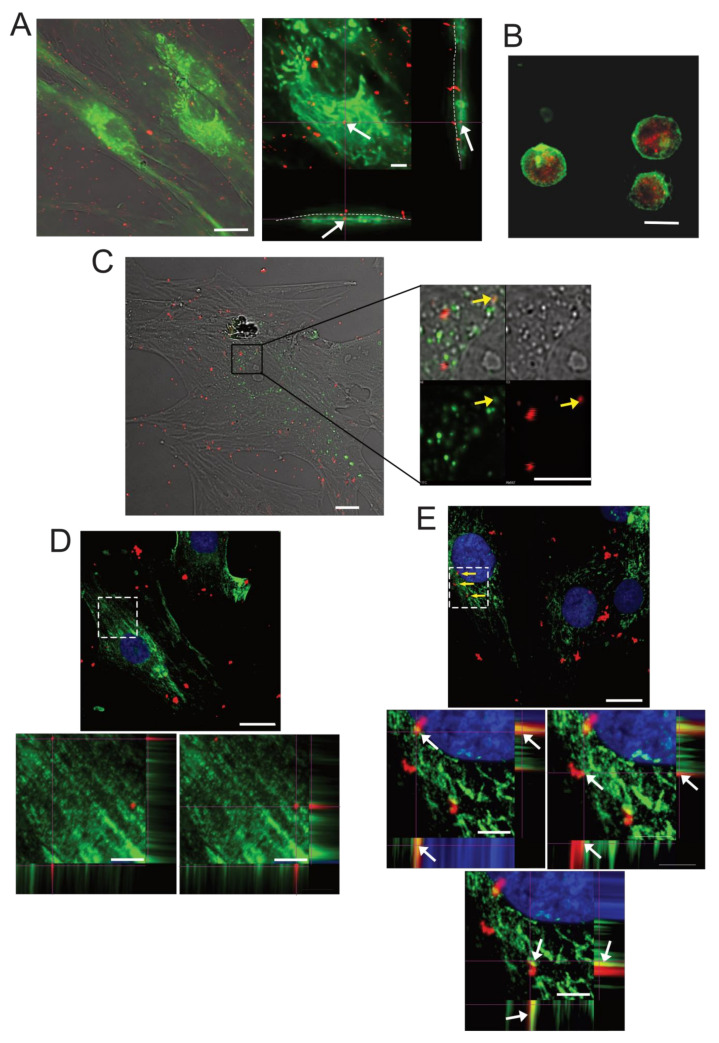
Internalization of SPN-curc-Cy5 into MSCs. (**A**) Representative image of confocal analysis of live BM-MSCs treated with SPN-curc-Cy5 4 h after incubation. The left panel shows the merge of transmitted light, green autofluorescence, and Cy5 emission (scale bar 20 μm). The right panel shows the optical xy, xz, and yz sections. The presence of SPN-curc-Cy5 in the green cytosol of the autofluorescent cell is highlighted by a white arrow (scale bar 5 μM). (**B**) BM-MSCs that were pretreated with SPN-curc-Cy5 and then detached and stained with PKH67 green fluorescent cell linker and observed as live cells (scale bar 20 μm). (**C**) Merge of transmitted light image of live BM-MSCs that were pretreated with SPN-curc-Cy5, and also incubated with LysoTracker-FITC (green) (scale bar 20 μm); an enlarged crop of an intracellular cytoplasmic area of the cell from the same image is also shown. Yellow arrows indicate the co-localization. (**D**) Immunofluorescence of fixed BM-MSCs that were treated prior to fixation with SPN-curc-Cy5. Merge of green (caveolin-1) and Cy5 emission (SPN-curc). Nuclei were counterstained with Hoechst 33258 (scale bar 20 μm). At the bottom, optical xy, xz, and yz sections of an enlarged crop of an intracellular cytoplasmic area of the cells from the same image are also shown (scale bar 5 μm). (**E**) Merge of green (LAMP3) and Cy5 emission (SPN-curc) in fixed BM-MSCs pretreated with SPN-curc-Cy5 and processed for immunostaining. Nuclei were counterstained with Hoechst 33258 (scale bar 20 μm). At the bottom, optical xy, xz, and yz sections of an enlarged crop of an intracellular cytoplasmic area of the cells from the same image are also shown (scale bar 5 μm). White arrows indicate the co-localization.

In order to determine the cellular uptake of SPN-curc into MSCs, particles were labelled with the near-infrared Cy5 fluorophore (SPN-curc-Cy5) (Figure 3C). BM-MSCs were incubated with SPN-curc-Cy5 (Figure 4) and observed via confocal microscopy, both as live adherent cells and as suspended cells. In adhesion, to enlighten the perimeter of the cells, we used both the autofluorescence of live cells and CD90 staining of fixed cells pre-treated with SPN-curc-Cy5. The z-stack images seem to suggest that MSCs had already efficiently internalized SPNs after 4 h of incubation (Figure 4A and Supplementary Video S1). By using CD90 staining, we could distinguish nanoparticles that were adherent to the cell membrane outside the cells (blue arrow, Supplementary Video S2) from nanoparticles that were untaken inside the cells (Supplementary Video S2, white arrows). However, since adherent cells were only 4 µm thick, we also performed an uptake assay on detached and suspended cells. By using a dye with a high affinity for lipids that binds to the cell membranes of live cells, it was clearly possible to distinguish the presence of several NP-curc-Cy5 in the intracellular and cytosolic space of the cells (Figure 1B, and Supplementary Video S3). Nanoparticles (red signal) appeared to be mostly located in intracellular vesicles. By performing the staining of lysosomes, endosomes, and late endosomes with LysoTracker, caveolin-1 [60], and the tetraspanin CD63/LAMP3 antibodies [61], respectively, we also showed that the accumulation of SPN-Cy5 mostly occurred in late endosomes (Figure 4C,D). Indeed, we found a colocalization of Cy5 signals mainly with the LAMP3 staining, to a lesser extent with LysoTracker staining (Figure 4C,E, yellow arrows), and none with caveolin-1 (Figure 4D). The extent of colocalization between caveolin-1 and SNP-curc-Cy5, or between CD63/LAMP3 and SNP-curc-Cy5, was also evaluated by analyzing the Pearson’s correlation coefficient (Supplementary Tables and Supplementary Figure S1). For CD63/LAMP3, for 5 ROIs out of 9 we assessed a value above 0.3, whereas for caveolin-1 the values were all negative. In conclusion, our results suggest that particles accumulate into the endo-lysosomal compartments.

### 2.4. Anti-Inflammatory Efficacy of SPN-curc

**Figure 5:**
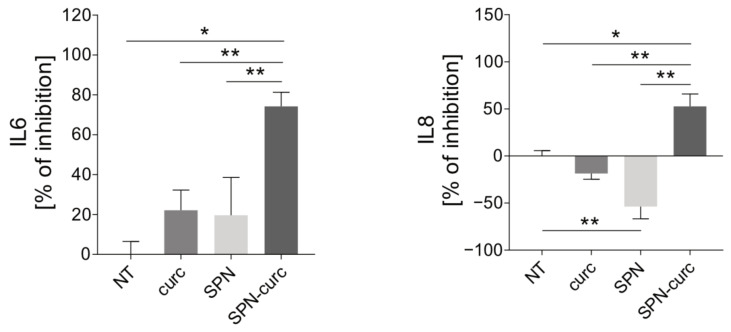
Effect of curcumin-loaded SPNs on the secretion of inflammatory cytokines by acid-treated MSCs. Results from ELISA quantification of IL6 and IL8 protein concentrations in the supernatants of acid-stressed MSCs under acidic conditions (pH 6.8), after 24 h of exposure to 4 µM free curcumin (curc), empty SPNs (SPN), or SPNs loaded with 4 µM curcumin (SPN-curc). Mean ± SE (*n* = 4, * *p* < 0.05 and ** *p* < 0.01).

We then verified the anti-inflammatory effectiveness of curcumin when loaded into SPNs. For this experiment, the amount of SPN-curc used was equivalent to the quantity needed in order to have a concentration of curcumin equal to 4 µM in the media. Empty SPNs were used as controls, in order to ensure that the vector itself produced no effect. SPN-curc displayed higher anti-inflammatory effects compared to the free curcumin (curc), since the inhibition activity was already significant at 4 µM (Figure 5, *p* = 0.0090 for both IL6 and IL8), suggesting that the curcumin’s encapsulation into SPNs improved its efficacy. The empty SPNs did not produce any significant effect on IL6 secretion. However, for IL8, we observed an increased release when the empty SPNs were used. This may indicate that the inhibitory effect observed with SPN-curc is solely due to the anti-inflammatory activity of curcumin.

### 2.5. Curcumin-Loaded SPNs Revert the Acid-Mediated, Pro-Tumorigenic Phenotype of MSCs

We next tested whether SPN-curc enhanced the inhibition of the pro-tumorigenic behavior of MSCs. To this aim, the stemness and invasiveness properties of OS were tested by performing a spherogenic assay [62,63] and a migration assay into a microfluidic chip, respectively. Specifically, HOS cells were co-cultured with BM-MSCs by using a transwell system for the spherogenic assay, or else exposed to BM-MSC supernatants for the migration test. As for the previous assays, the stressful stimulus of the acidic TME was reproduced by culturing BM-MSCs in buffered media at pH 6.8. In both assays, BM-MSCs were pre-exposed to acidosis, and to four different experimental conditions: (1) no treatment (pH 6.8); (2) treatment with free curcumin (curc); (3) treatment with empty SPNs (SPN); and (4) treatment with SPN-curc (SPN-curc). 

**Figure 6:**
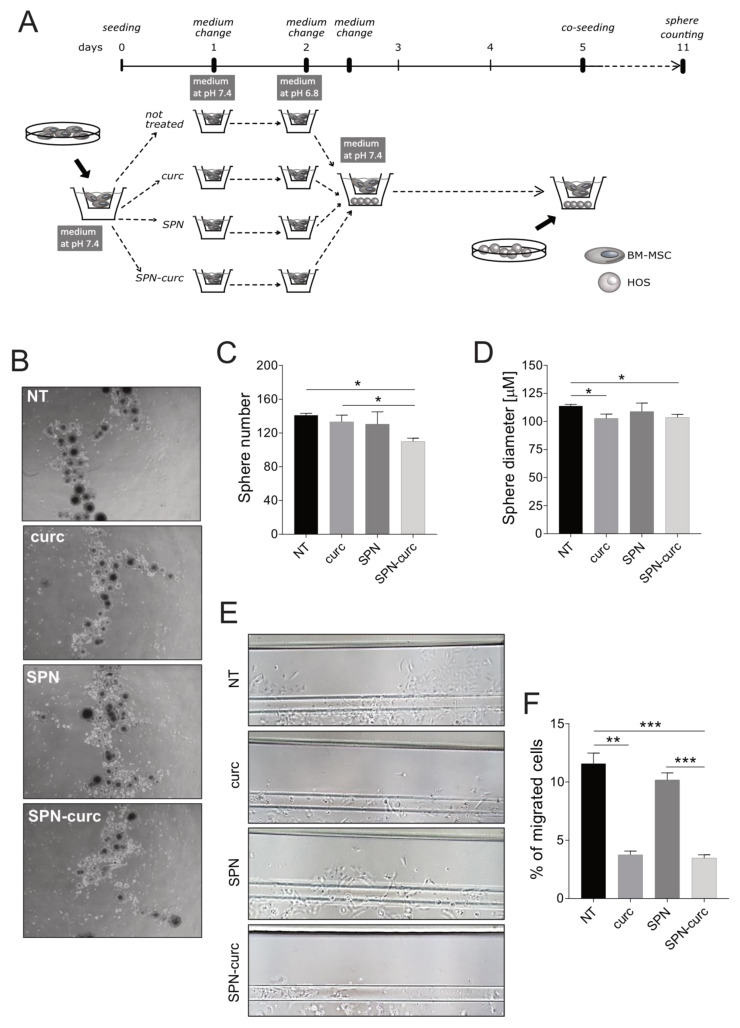
SPN-curc impaired the pro-tumorigenic activity of MSCs induced by acidosis. (**A**) Experimental scheme of the transwell co-culture system used for the sphere-forming efficiency assay. BM-MSCs were previously stressed with short-term acidosis, and either treated, or not (NT), with 4 µM free curcumin (curc), empty SPNs (SPN), or SPNs loaded with 4 µM curcumin (SPN-curc). The co-culturing of HOS cells with BM-MSCs was prolonged for 6 days (see Section 4.8. for further details). (**B**) Representative images of the HOS spheres obtained after co-culturing with BM-MSCs. (**C**) Quantification of the number of HOS spheres. Mean ± SE (*n* = 6, * *p* < 0.05). (**D**) Quantification of HOS spheres’ diameter. Mean ± SE (*n* = 6, * *p* < 0.05). (**E**) Representative images of migrated HOS cells in 3D microfluidic chips after exposure for 72 h to MSC supernatants. MSCs were pre-treated with acidic medium, and either treated, or not (NT), with 4 µM free curcumin (curc), empty SPNs (SPN), or SPNs loaded with 4 µM curcumin (SPN-curc). (**F**) Percentage of migrated and invaded cells, expressed as the ratio between the number of HOS cells that were migrated to the upper lane and the total number of HOS cells within the microfluidic device x 100. Mean ± SE (*n* = 6, ** *p* < 0.01, and *** *p* < 0.001).

Spherogenic assay is the most widely accepted method of isolating cancer stem cells (CSCs)—as we also demonstrated for sarcoma cells [64]—and, as an index of stemness, measures their ability to grow as floating spheres in the absence of fetal serum. To evaluate the effect of SPN-curc on HOS spherogenicity, we co-cultured HOS cells and acid-stressed BM-MSCs, either treated, or not (NT), with 4 µM free curcumin (curc), empty SPNs (SPN), or SPNs loaded with 4 µM curcumin (SPN-curc), by using a transwell system, as schematically described in Figure 6A. As shown in the representative images (Figure 6B), treatment of MSCs with SPN-curc significantly reduced the number of OS spheres (*p* = 0.0495, Figure 6C). Of note, SPN-curc treatment was also more effective in decreasing the number of spheres than free curcumin, which, at the concentration used, was ineffective (*p* = 0.0495, Figure 6C). SPN-curc and free curcumin also significantly reduced the cancer sphere diameter (*p* = 0.0495, Figure 6D). Furthermore, the increased IL8 secretion recorded after MSC treatment with SPNs did not affect OS cell stemness.

To assess OS cell migration and invasion, we used a 3D microfluidic chip and a passive perfusion system to recapitulate the perfusion of the TME in vivo [65,66]. We seeded Matrigel-embedded HOS cells in one of the two channels of the chip, as schematically shown in Supplementary Figure S2A. We then exposed the HOS cell-enriched matrix to the supernatants of MSCs treated, or not, with curcumin, SPN-curc, or empty SPNs. As an index of the migration and invasion ability of tumor cells, the number of cells that were able to degrade the extracellular matrix and migrate into the adjacent Matrigel-free channel was counted. As shown in the representative images (Figure 6E), HOS cells exposed to the supernatant of MSCs pre-incubated with SPN-curc showed a significantly lower ability to degrade the surrounding matrix and migrate (Figure 6F, *p* = 0.0006 vs pH 6.8), compared to control. Similar results were found for curcumin-treated cells, while empty SPNs did not produce any change with respect to the untreated condition. The inhibitory effect of SPN-curc was also confirmed in the 143b and Saos-2 osteosarcoma cell lines (Supplementary Figure S2B and Figure 2C, respectively, *p* = 0.0495 vs. pH 6.8). 

## 3. Discussion

The anti-proliferative and pro-apoptotic effects of curcumin on cancer cells have been extensively described in several solid tumors [67], including OS [41,42]. In contrast, only limited data are available on the potential of curcumin to affect the pro-tumor properties of cancer-associated stromal cells. However, cancer growth and progression are largely dependent on the reciprocal interaction between cancer cells and the stroma [1], with particular reference to MSCs, as these multipotent stem cells are prone to react to stressful microenvironmental stimuli, including local acidosis. In OS, we have shown that, in the presence of the altered acidic TME, MSCs secrete a plethora of inflammatory mediators that further exacerbate OS malignancy through NF-κB activation [21]. We therefore hypothesized that curcumin, as a NF-κB blocker, has the potential to combine anti-inflammatory and anti-tumor effects on cancers such as OS, with the advantages of high accessibility, cost-effectiveness, and safety [31]. 

First, we confirmed data from the literature [56,68] showing that cancer cells are more sensitive to the cytotoxic activity of curcumin than their normal counterparts under both neutral and acidic conditions. We chose MSCs as controls because accumulating evidence has placed MSCs and/or their immediate lineage progenitors as the most likely cells-of-origin for several types of sarcomas, including OS [69]. Evaluation of curcumin efficacy under acidic conditions is useful because microenvironmental chemical factors, such as acidosis, can strongly influence the stability and therapeutic efficacy of drugs [70], and represent an important pharmacological concern [71]. However, in our model, the acidic TME did not significantly affect the selective cytotoxicity of curcumin to cancer cells. In this regard, it is noteworthy that intratumoral acidosis activates cIAP/TRAF/NF-κB signaling in OS cell lines [72], suggesting that this pro-survival pathway can be effectively and advantageously targeted by curcumin. Activation of the NF-κB pathway mediates the increase of several anti-apoptotic genes and mitogenic proteins in tumor cells that indirectly impact on cancer cell survival, growth, and metastasis [73]. In addition, curcumin is able to inhibit additional pathways other than NF-κB, including cell proliferation (cyclin D1, c-myc), cell survival (Bcl-2, Bcl-xL, cFLIP, XIAP, and c-IAP1), and protein kinase pathways (JNK, Akt, and AMPK) [56]. 

We therefore focused on the use of curcumin as an anti-inflammatory drug to affect the acid-induced and pro-tumor activity of MSCs. We assessed the nuclear internalization of the RelB protein as a marker of the activation of the NF-κB inflammatory pathway, and the secretion of IL6 and IL8 as a downstream effect of the NF-κB pathway, as these cytokines are significantly involved in cancer progression [74,75], and are strongly increased after exposure to an acidic medium. First, curcumin treatment significantly affected RelB nuclear translocation. Conversely, no inhibitory effect was recorded during treatment of MSCs with the lowest concentration of free curcumin (4 µM)—neither for IL6 nor for IL8 secretion. In contrast, curcumin concentrations equal to or greater than 10 µM were effective in inhibiting IL6 and IL8 secretion by MSCs reprogrammed by acidosis. Interestingly, as previously demonstrated by the cell viability assay, the lowest doses (10 and 20 µM) that were effective in reducing the secretion of both ILs were not cytotoxic to MSCs, implying that the anti-inflammatory activity can be achieved at nontoxic concentrations of the drug. We also assessed the impact of curcumin on the secretion of additional cytokines that are activated by the NF-κB pathway. In particular, we found that TGF-β1 secretion was significantly inhibited by curcumin treatment in a dose-dependent manner, and with a strong effect even at the 4 µM concentration. The lowest concentration of the drug (4 µM) was also sufficient to reduce IL12 secretion by BM-MSCs.

Before proceeding with the experiments, we considered that in order to employ curcumin as a therapeutic strategy, several obstacles must be overcome—including low bioavailability, poor absorption, and rapid metabolism—which can only be resolved by increasing the dosage, with associated toxic effects [45]. To overcome these critical issues, in our previous studies, we encapsulated curcumin in novel lipid-based spherical polymeric nanoparticles (SPN-curc). We have already shown that encapsulation of the drug in SPNs is a valuable therapeutic approach, as it improves the targeting and efficacy of curcumin, even at low concentrations [49], on a variety of cell models. For example, we recently demonstrated its efficacy in modulating the vascular deposition of CTCs in a highly metastatic breast cancer cell line [51], and in counteracting inflammation stimulated by amyloid-β fibers [53]. We then verified the successful delivery and internalization of SPN-curc into MSCs by labelling the nanoparticles with a far-red fluorophore, as we have previously done with other cell types [53,76,77,78]. The fluorescence signal was mostly distributed in the perinuclear regions of cells, similar to cancer and endothelial cells [51]. In addition, after curcumin was loaded into the SPNs, it effectively reduced IL6 and IL8 secretion at the lowest concentration tested in this study (4 µm) that was not effective as a free drug, suggesting that encapsulation in lipid-polymeric nanoparticles improved drug efficacy.

IL6 secreted by MSCs is an important mediator of OS cell stemness, migration, and invasion. More specifically, IL6 secretion by acid-reprogrammed MSCs further expands the CSC subpopulation of OS, and induces cancer cell migration [21]. CSCs are a small subset of tumor cells with stem-like features that are responsible, based on their self-renewing ability and competence, for giving rise to differentiated progeny, both at tumor initiation and at the development of local and systemic relapse [79]. CSCs have also been isolated from OS [64]. Because curcumin-loaded SPNs efficiently decreased the acid-induced IL6 secretion by MSCs, we wondered whether they were also able to impair IL6-triggered OS stemness and invasion. We then used the spherogenic assay to measure the stemness potential of OS cells [64]. We found that MSC treatment with SPN-curc strongly impaired the stem-like phenotype of OS cells promoted by the acid-induced inflammatory reaction, confirming that targeting the tumor-associated and stromal cells subjected to acidic stress by curcumin-loaded nanoparticles can effectively prevent the induction of OS stemness.

Finally, we further dissected the pharmacological efficacy of SPN-curc in reversing the pro-tumorigenic behavior of MSCs subjected to acid stress. To this end, we used a microfluidic model to simulate the local in vivo invasion of OS cells. Such a platform allows for the integration of perfusion flow, gradients (e.g., chemotaxis), and mechanical stresses, and thus in an in vitro environment it mimics for crucial aspects of cancer progression, such as migration and invasion [80]. The microfluidic model confirmed the efficacy of SPN-curc in reducing MSC-stimulated OS migration and invasion. Of note, although treatment with empty SPNs increased IL8 secretion by MSCs, it did not affect the stemness or invasiveness properties of OS cells when co-cultured with pre-treated MSCs, indicating that SPNs’ effects are possibly transitory and, in any case, does not worsen tumor features.

Overall, our data demonstrate that incorporation into lipid-based nanosystems significantly enhances the curcumin-mediated blockade of the inflammatory reaction of MSCs responsible for their pro-tumor phenotype, even at very low concentrations of the drug, and suggest their use for the treatment of acidifying cancers. Finally, it should be emphasized that the proposed nanoparticles can be readily modified to include targeting moieties to enhance specificity, additional therapeutic agents for multi-drug therapies [81], and contrast agents for real-time imaging [82].

In this work, we therefore effectively developed and validated a pharmacological strategy that combines the direct cytotoxic effects of curcumin on tumor cells with its potential to counteract the inflammatory reaction to acidosis of the stromal counterpart of OS, with the ultimate goal of impairing tumor progression.

## 4. Materials and Methods 

### 4.1. Cell Cultures

Human MSCs from bone marrow (BM-MSCs) were purchased from Lonza (Basel, Switzerland, catalogue *n*. PT-2501, lot #6F4085) and cultured in Minimum Essential Medium Eagle Alpha Modified (Alpha-MEM) (Sigma-Aldrich, St. Louis, MO, USA) with 100 U/mL penicillin, 100 mg/mL streptomycin, and 10% FBS (Euroclone, Milan, Italy), and buffered at pH 7.4 (complete) or 0.1% FBS (low-serum). BM-MSCs were always used before the 5–6 passage in culture. The human osteosarcoma cell lines HOS, 143b, and Saos-2 were purchased from ATCC and cultured in Iscove’s Modified Dulbecco’s Medium (IMDM) (Life Technologies, Carlsbad, CA, USA) with 100 units/mL penicillin, 100 mg/mL streptomycin, and 10% FBS (complete IMDM). Cells were maintained at 37 °C, 5% CO_2_ in a humidified atmosphere. Culture media at specific pH levels were obtained by adjusting the concentration of sodium bicarbonate according to the Henderson–Hasselbach equation [83]. When not differently specified, acidic media were buffered at pH 6.8, and physiological media were buffered at pH 7.4. At different timepoints and at the endpoint of each experiment, the pH of the supernatant was always measured in order to ascertain the maintenance of the pH value along the incubation time.

### 4.2. Nanoparticle Synthesis and Characterization

#### 4.2.1. Materials

Poly(D, L-lactide-co-glycolide) acid-terminated (PLGA, lactide:glycolide 50:50, Mw 38,000–54,000), was purchased from Sigma-Aldrich (St. Louis, MO, USA). Curcumin (Cur) was bought from AlfaAesar (Haverhill, MA, USA). 1,2-distearoyl-sn-glycero-3-phosphoethanolamine-N-[succinyl(polyethylene glycol)-2000] (DSPE-PEG-COOH) and 1,2-Dipalmitoyl-sn-glycero-3-phosphocholine (DPPC) were purchased from Avanti Polar Lipid (Alabaster, AL, USA). All reagents and solvents were used without further purification.

#### 4.2.2. Synthesis of SPNs 

Spherical polymeric nanoparticles (SPN) were synthetized using a sonication–emulsion method, as described elsewhere [58]. Briefly, an oil phase containing carboxyl-terminated poly(lactic-co-glycolic acid) (PLGA), 1,2-dipalmitoyl-sn-glycero-3-phosphocholine (DPPC), and Curcumin (Curc) was added drop by drop to an aqueous phase containing 1,2-distearoyl-sn-glycero-3-phosphoethanolamine-N-[carboxy(polyethylene glycol)-2000 (DSPE-PEG-COOH) in 4% ethanol, under sonication (100% amplitude for 1.5 min). The so-formed emulsion was magnetically stirred for 3 h to facilitate the organic solvent evaporation. Then, SPNs were centrifuged, first for 5 min at 1500 rpm in order to settle down any possible debris, and then the supernatants were centrifuged 3 more times for 20 min at 12,000 rpm. The pellets were washed in water after every centrifugation step. For the fabrication of empty SPNs (SPNs), the same procedure was followed, but curcumin was not added to the oil phase. For confocal microscopy imaging, SPNs were labelled using DSPE-Cy5 (synthetized as indicated elsewhere [84]). For the fabrication of SPN-curc-Cy5, 0.002 mg of DSPE-Cy5 was added in the aqueous phase and the same procedure used for the fabrication of curc-SPN was followed.

#### 4.2.3. Characterization of SPNs 

A drop of SPNs was deposited on a silicon wafer, dried, and mounted on a stab for SEM analysis. SEM images were collected using JEOL JSM-7500FA (Jeol, Tokyo, JAPAN) operating at 5 kV of accelerating voltage. The hydrodynamic diameter, polydispersity index, and surface electron ζ-potential of the SPNs were measured using dynamic light scattering (DLS, Malvern Zetasizer Nano S). 

#### 4.2.4. Drug Loading and Release 

To measure the Curc encapsulation efficiency (*EE*), samples were dried, dissolved in acetonitrile, and their absorbance analyzed at 420 nm by spectrophotometer (Tecan, Männedorf, Swiss). *EE* was determined using the following Equation:

(1) $$EE\;\left(\%\right)=\frac{\mathrm{Cur}\;\mathrm{weight}\;\mathrm{in}\;\mathrm{particles}}{\mathrm{Cur}\;\mathrm{initial}\;\mathrm{feeding}\;\mathrm{amount}}\times 100$$

To study Curc-release kinetics, 200 μL of SPN-curc solution were placed into Slide-A-Lyzer MINI dialysis microtubes, with a molecular cutoff of 10 kDa (Thermo Scientific, Waltham, MA, USA), and dialyzed against 4 L of PBS buffer (pH 7.4 or 6.8) or acetate buffer (pH 5.0). For each timepoint, three samples were collected and dried. For the SPNs, samples were then dissolved in acetonitrile and analyzed by spectrophotometer.

### 4.3. Cellular Uptake

BM-MSCs were seeded into a 35 mm diameter glass petri dish in complete Alpha-MEM. After reaching 70% confluence, cells were incubated with fresh complete Alpha-MEM containing the SPNs. In order to trace the nanoconstructs and to ascertain their internalization into MSCs, SPN-curc were labeled with the red fluorophore Cy5 (GE Healthcare, Little Chalfont, Buckinghamshire, England) (SPN-curc-Cy5). Cellular uptake was monitored during 8 h of treatment using confocal microscopy (Z-Step 0.825 µM, loop interval 7 min, A1R, Nikon, Minato, Tokyo, Japan) on both adherent and suspended live cells. For live adherent cells, the green autofluorescence was detected by using 487.2 laser power at 2.6, PMT HV 67, PMT Offset 7, pinhole size 42.15 µm, scan speed 7.5, resonant scanning, zoom at 1, and line average of 4 scans, and post-acquisition LUTs adjustment 110–259. Z-Step corresponded to 0.82 µM, 39 Z-stack loops with Ni-E ZDrive. Objective 40x water, numerical aperture 0.8. For suspended cells, after treatment with SPN-curc-Cy5, cells were collected by trypsinization and then stained with a green fluorescent cell linker dye (PKH67, Sigma-Aldrich, St. Louis, MO, USA) in order to stain cell membranes. The fluorescent staining was then acquired by confocal microscopy (A1R, Nikon, Minato, Tokyo, Japan) using objective 20x air, numerical aperture 0.75, galvano scanning, zoom at 3.205, and line average of 4 scan.

### 4.4. Lysotracker Staining

Living adherent cells at low confluence were maintained in complete medium at 37 °C and 5% CO_2_ and treated for 4 h with SPN-curc-Cy5 at 4 µM concentration. After treatment, medium was replaced with fresh medium added with LysoTracker™ Green DND-26 (50 nM, Molecular Probes, Life Technologies, Carlsbad, CA, USA). At the end of the incubation period, medium was replaced again and cells were then analyzed using a confocal microscope (A1R, Nikon, Minato, Tokyo, Japan) with objective 20x air, numerical aperture 0.75, galvano scanning, zoom at 2.639, and line average of 4 scans).

### 4.5. Immunofluorescence

Adherent cells were fixed with 3.7% paraformaldehyde and analyzed for immunostaining. Fixed cells were preliminarily permeabilized with 0.1% Triton X-100 and incubated with 1% BSA, followed by primary antibodies diluted in 0.1% BSA for 1 h, at 4 °C—rabbit anti-RelB (HPA040506, Sigma-Aldrich, St. Louis, MO, USA), rabbit anti-CD63/LAMP3 (sc-15363, Santa Cruz Biotechnology Inc., Dallas, TX, USA), and rabbit anti-caveolin-1 (610059, Transduction Laboratories BD, Franklin Lakes, NJ, USA). As a secondary antibody, we used an anti-rabbit antibody conjugated with Alexa Fluor 488 nm (A11008, Life Technologies, Carlsbad, CA, USA). Nuclei were stained with Hoechst 33258. The fluorescence signal was then detected using a confocal microscope (Z-Step 0.225 µM, A1R, Nikon, Minato, Tokyo, Japan), and with the following acquisition parameters. For RelB: objective 20x air, numerical aperture 0.75, galvano scanning, zoom at 1, line average of 2; for caveolin-1: Z-Step 0.23 µM, 7 Z-stack loops with Ni-E ZDrive, objective 60x oil, numerical aperture 1.4, galvano scanning, zoom at 1.869, line average of 2 scans: for CD63/LAMP3: Z-Step 0.23 µM, 5 Z-stack loops with Ni-E ZDrive, objective 60x oil, numerical aperture 1.4, galvano scanning, zoom at 1.869, line average of 2 scans. Co-localization analysis between Cy5 signals and CD63/LAMP3 or caveolin-1 was evaluated using Pearson’s correlation coefficient (NIS-Elements Microscope Imaging Software, Nikon, Minato, Tokyo, Japan), on 5–7 different z-steps of 0.25 µm (see Supplementary Tables).

For CD90 immunofluorescence, cells were fixed with 3.7% paraformaldehyde and incubated with CD90-FITC conjugated antibody (IM1839U, Beckman Coulter Inc., Brea, CA, USA) diluted in 0.1% BSA for 40 min. The fluorescence signal was then detected using a confocal microscope (A1R, Nikon, Minato, Tokyo, Japan).

For the quantification of the nuclear signal or RelB, we automatically detected the area of the nuclei by using NIS-Elements Microscope Imaging Software (Nikon, Minato, Tokyo, Japan). We automatically detected ROIs, based on the emission of Hoechst 33258. We then evaluated the mean intensity of the red signal associated with RelB. As a result, for the nuclear RelB signal quantification, we considered the ratio between the mean intensity of the red signal in the nuclear area and the nuclear area (ROI area).

### 4.6. Cytotoxicity Assay

To calculate the IC_50_ values for free curcumin in neutral and acidic conditions, cells were seeded into 96-well plates (4 × 10^3^ cells/well for HOS, and 3.2 × 10^3^ for BM-MSC) in complete IMDM. After 24 h, the medium was changed, with complete medium buffered at pH 7.4 or pH 6.8, added with curcumin (0–4–10–20–50–100–250 µM) or vehicle (DMSO at the same percentage used to make curcumin dilutions, that was in any case < 0.3%).

After 48 h since the addition of curcumin, the cell viability was measured using an acid phosphatase assay, as previously described [85]. Briefly, the culture medium was removed, cells were washed with PBS, and 100 μL of buffer containing 0.1 M sodium acetate (pH 5.0), 0.1% Triton X-100, and 5 mM p-nitrophenil phosphate (Sigma-Aldrich, St. Louis, MO, USA) were added. After 3 h of cell incubation at 37 °C, the reaction was stopped with the addition of 10 μL of a 1 N NaOH solution, and color development was recorded at 405 nm using a microplate reader (Tecan Infinite F200pro, Tecan, Männedorf, Zurich, Switzerland). Data were expressed as the percentage of cell viability obtained as the ratio between OD measured in cells exposed to compounds and OD measured in negative control (cells cultured in medium not added with curcumin at the specific pH) x 100. The drug half-maximal inhibitory concentration (IC_50_) was calculated by linear regression using the Graph Pad Prism 7.04 software. The experiment was performed twice in quadruplicate.

### 4.7. ELISA

In order to assess the protein concentration of pro-inflammatory cytokines in the cell supernatants, BM-MSCs were seeded into a 24-well plate (3 × 10^4^ cells/well) in complete alpha-MEM. After adhesion, cells were pre-treated for 24 h with low-serum alpha-MEM at pH 7.4—combined, or not, with different experimental conditions, depending on the aim of the assay. To determine the minimum effective dose of free curcumin that is able to block acid-induced cytokine secretion by MSCs, cells were exposed to increasing concentrations of free curcumin (0–4–10–20–50 µM). To evaluate the effect of curcumin encapsulation into SPNs on the inhibitory efficacy of the drug, cells were exposed to SPNs loaded with 4µM curcumin (SPN-curc), 4µM free curcumin (curc), and empty SPNs (SPN).

After the cell pre-treatment performed in low-serum neutral medium in order to ensure cell adaptation to serum deprivation, BM-MSCs were treated for an additional 24 h with the same variety of experimental conditions described above in low-serum Alpha-MEM at pH 6.8, in order to mimic the acidic stress responsible for the NF-κB activation and for the downstream inflammatory reaction of MSCs, which are thought to support OS progression. 

In all of the experiments, the negative control for the inhibition of cytokine secretion was the low-serum Alpha-MEM at acidic pH without any treatment. For the experiments with SPNs, the low-serum Alpha-MEM at pH 6.8 combined with free curcumin (curc) and with empty SPNs (SPN) were considered, respectively, to be positive and negative controls of the pharmacological effectiveness of the proposed nanosystem. 

After the short-term exposure to low pH, the MSC supernatants were collected, centrifuged at 1600 rpm in a cold refrigerated centrifuge (+4 °C) for 10 min, aliquoted, and stored at −80 °C for further analysis of their cytokine content. 

IL6 and IL8 contents were determined in the MSC supernatants by using the Human IL6 DuoSet ELISA and the Human CXCL8/IL8 Quantikine ELISA Kits (R&D Systems, Minneapolis, MN, USA), respectively, according to the manufacturer’s instructions. IL-1β, TNF-α, TGF-β1, and IL12 contents were determined in MSC supernatants by using the Human IL-1 beta/IL-1F2, the Human TNF-alpha, the Human TGF-beta 1, and the Human IL-12 p70 Quantikine ELISA Kits (R&D Systems, Minneapolis, MN, USA), respectively. For all of the ELISA assays, protein concentration was normalized to the total protein content quantified by the BCA assay. The experiments were performed twice in duplicate.

### 4.8. Sarcosphere-Forming Efficiency

To evaluate the effect of BM-MSC treatment with SPN-curc on OS spherogenicity, we performed a co-culture of BM-MSC and HOS cells using transwell inserts with a 0.4 µm porous membrane. BM-MSCs were seeded into the transwell inserts (1.4 × 10^4^ cells/transwell) in complete alpha-MEM. After adhesion, cells were pre-treated for 24 h with SPNs loaded with 4 µM curcumin (SPN-curc), 4 µM free curcumin (curc), and empty SPNs (SPN). After 1 wash with PBS, the pharmacologic treatment was then repeated in low-serum medium at pH 6.8 for additional 10 h. The medium was then replaced with low-serum medium at pH 7.4. After an additional 48 h, HOS cells were seeded (2 × 10^4^ cells/well) in DMEM:F12 medium at pH 7.4 with progesterone (20 nM), putrescine (10 mg/mL), sodium selenite (30 nM), apo-transferrin (100 µg/mL), and insulin (25 µg/mL) in low-attachment 12-well plates, and the transwell inserts containing MSCs were placed over these wells. After 6 days of co-culture, the total number and the sphere diameter of the HOS spheres was counted and quantified by using NIS-Elements Microscope Imaging Software (Nikon). The experiment was performed twice in triplicate.

### 4.9. Cell Invasion Assay in Microfluidic Devices

In order to collect the MSC-conditioned medium (MSC CM) used to test OS migration and invasion in microfluidic devices, BM-MSCs at the 4^th^–5^th^ passages were seeded in T25 cell culture flasks (3 × 10^5^ cells/flask) in complete Alpha-MEM. After adhesion, cells were pre-treated, or not pre-treated, with SPN-curc, curc, or SPNs for 24 h in low-serum medium at pH 7.4, washed with PBS, and incubated with the same experimental conditions for 10 h in low-serum medium at pH 6.8. At the end of the acidic stress, cells were washed twice with PBS in order to remove any residual nanoparticles, and incubated with low-serum medium at pH 7.4 for an additional 48 h for all of the conditions. This pH-neutral supernatant, which we named MSC CM, was finally collected, centrifuged at 1600 rpm in a cold refrigerated centrifuge (+4 °C) for 10 min to remove cell debris, and stored at −80 °C until use.

We used 400 μm 2-lane OrganoPlates^®^ consisting of 96 microfluidic chips (Mimetas B.V., Leiden, The Netherlands) as microfluidic devices in order to assess cell migration and invasion in response to MSC CM.

Before gel seeding, every center well was filled with 50 µL of PBS in order to provide optical clarity and prevent gel dehydration. Using a repeater pipette, 1.5 µL of the 1:2 diluted Matrigel (BD Biosciences, Franklin Lakes, NJ, USA) containing osteosarcoma cells (2 × 10^3^ for HOS, 1.8 × 10^3^ for 143b, and 1.5 × 10^3^ for Saos-2 cells) was added to the inlet of each gel channel. To ensure polymerization of the Matrigel, the device was incubated for 15 min at 37 °C, 5% CO_2_ in a humidified atmosphere. After incubation, 50 µL of MSC-CM obtained as previously described was added to the perfusion inlet and outlet wells, and the plates were placed on an interval rocker platform for continuous perfusion (Perfusion rocker, Mimetas B.V., Leiden, The Netherlands). The rocker was set at a 7-degree inclination and 8-min cycle time, and placed at 37 °C, 5% CO_2_ in a humidified atmosphere. After 72 h, the cells that were migrated towards the upper lane of each microfluidic chips were counted (20 × lens). Data were expressed as the percentage of migrated cells, considered as the ratio between the number of migrated cells and the total number of cells counted in the microfluidic device × 100. The experiment was performed twice in triplicate.

### 4.10. Statistical Analysis

Statistical analysis was performed using the Graph Pad Prism 7.04 software for Windows (Graph Pad Software, La Jolla, CA, USA). Because of the small number of observations, data were not considered to be normally distributed and, therefore, non-parametric tests were used. The Mann–Whitney U test was used as an unpaired comparison for two independent variables. Data were expressed as mean ± standard error (SE). Only *p* values < 0.05 were considered for statistical significance.

## Data Availability

Not applicable.

## Supplementary Materials

The following are available online at https://www.mdpi.com/article/10.3390/ijms22115760/s1.

## Abbreviations

TME	tumor microenvironment
CAFs	cancer-associated fibroblasts
MSCs	mesenchymal stromal cells
OS	osteosarcoma
RelA	RelA proto-oncogene NF-κB Subunit
RelB	RelB proto-oncogene NF-κB subunit
NFKB1	nuclear factor kappa B subunit 1
IκBα	nuclear factor of kappa light polypeptide gene enhancer in B-cells inhibitor alpha
SPN	spherical polymeric nanoparticles
SPN-curc	spherical polymeric nanoparticles encapsulating curcumin
CTCs	circulating tumor cells
BM-MSC	bone marrow-derived MSCs
IL6	interleukin 6
IL8	interleukin 8
IL-1β	interleukin 1beta
TNF-α	tumor necrosis factor alpha
TGF-β1	transforming growth factor beta 1
IL12	interleukin 12
PLGA	poly(lactic-co-glycolic acid)
DPPC	1,2-dipalmitoyl-sn-glycero-3-phosphocholine
curc	curcumin
SPN-curc-Cy5	spherical polymeric nanoparticles encapsulating curcumin and labelled with Cy5 fluorophore
CSCs	cancer stem cells
