# Supplementary material for: Curcumin-Loaded Nanoparticles Impair the Pro-Tumor Activity of Acid-Stressed MSC in an In Vitro Model of Osteosarcoma
Source: Int J Mol Sci. 2021 May 28;22(11):5760. doi: 10.3390/ijms22115760 (PMC8198446; doi:10.3390/ijms22115760)
Supplement: Supplementary file 1 [file ijms-22-05760-s001.zip › Supplementary Figures revised.pdf]

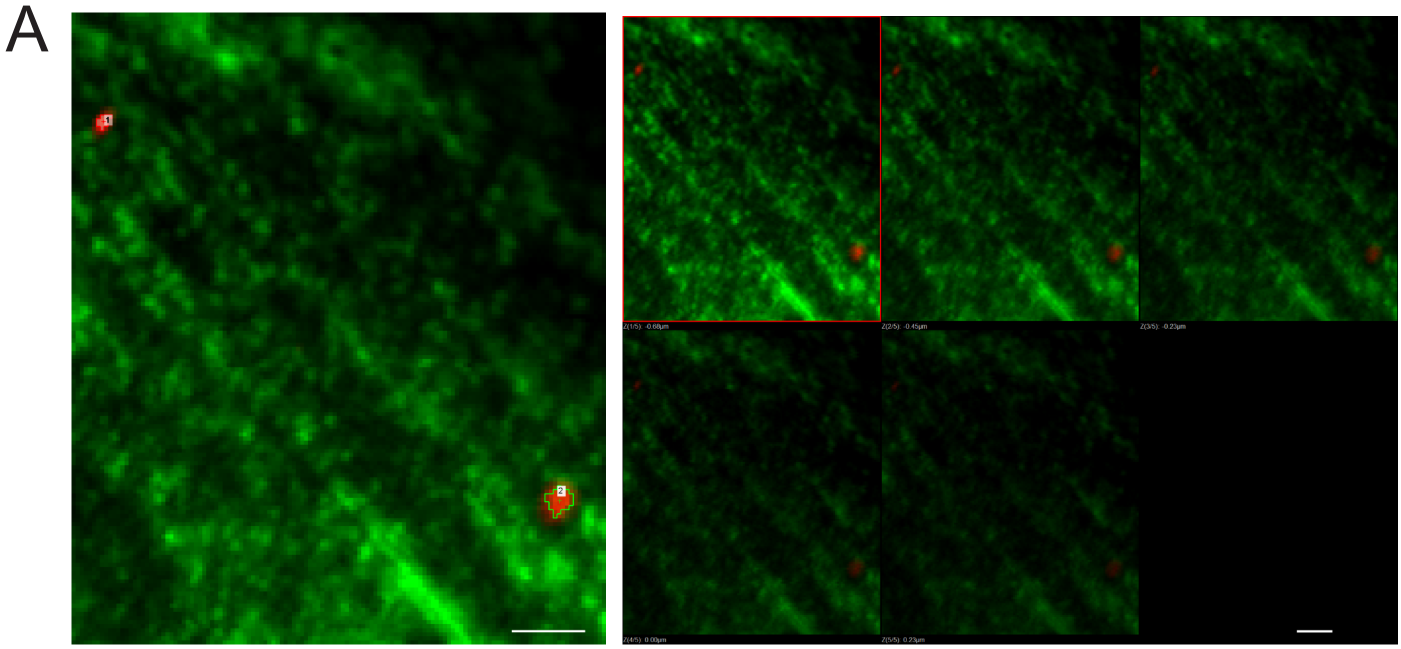

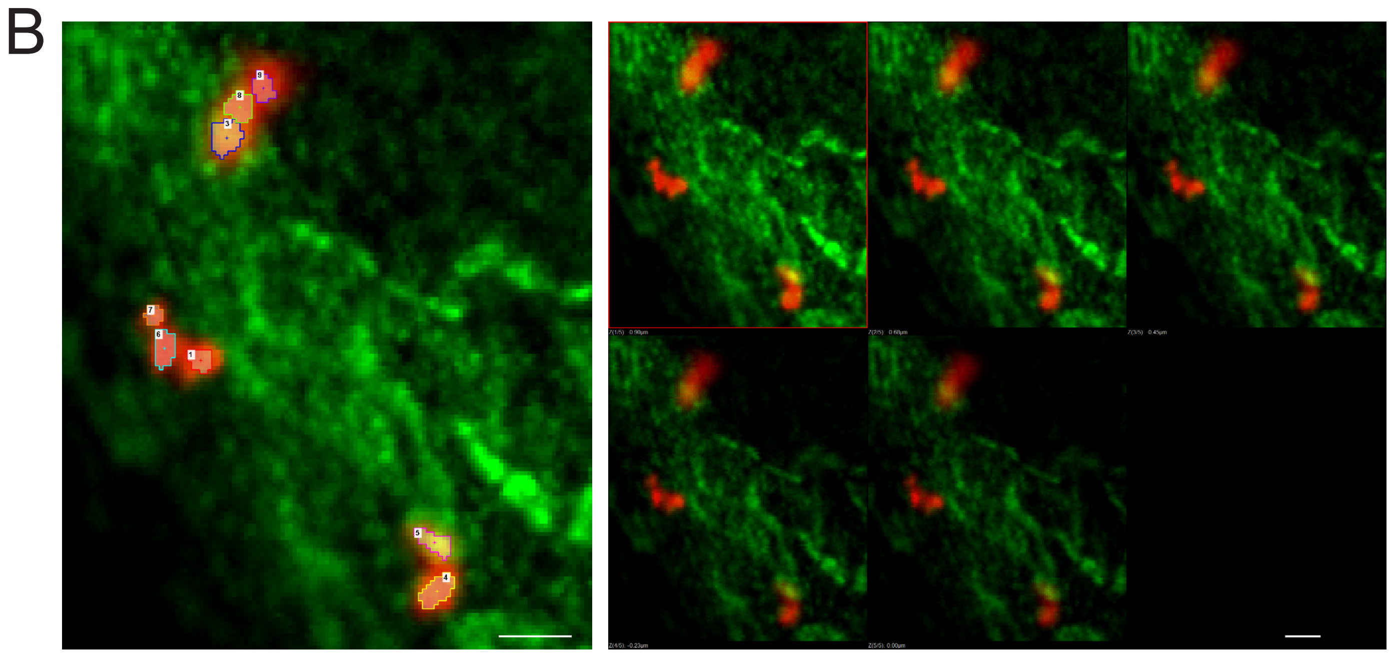

Figure S1. Co-localization analysis of SPN-curc-Cy5 and lysosomal-endosomal markers in MSCs. (**A**) On the left panel, representative image of the ROIs examined for the analysis of the co-localization between caveolin1 (FITC) and SPN-curc (Cy5) signals, and on the right panel, the five z-steps of 0.225 μM that were considered for the analysis (scale bar 2 μm); (**B**) On the left panel, representative image of the ROIs examined for the analysis of the co-localization between the CD63/LAMP3 (FITC) and SPN (Cy5) signals, and on the right panel, the five z-steps of 0.225 μM that were considered for the analysis (scale bar 2 μm).

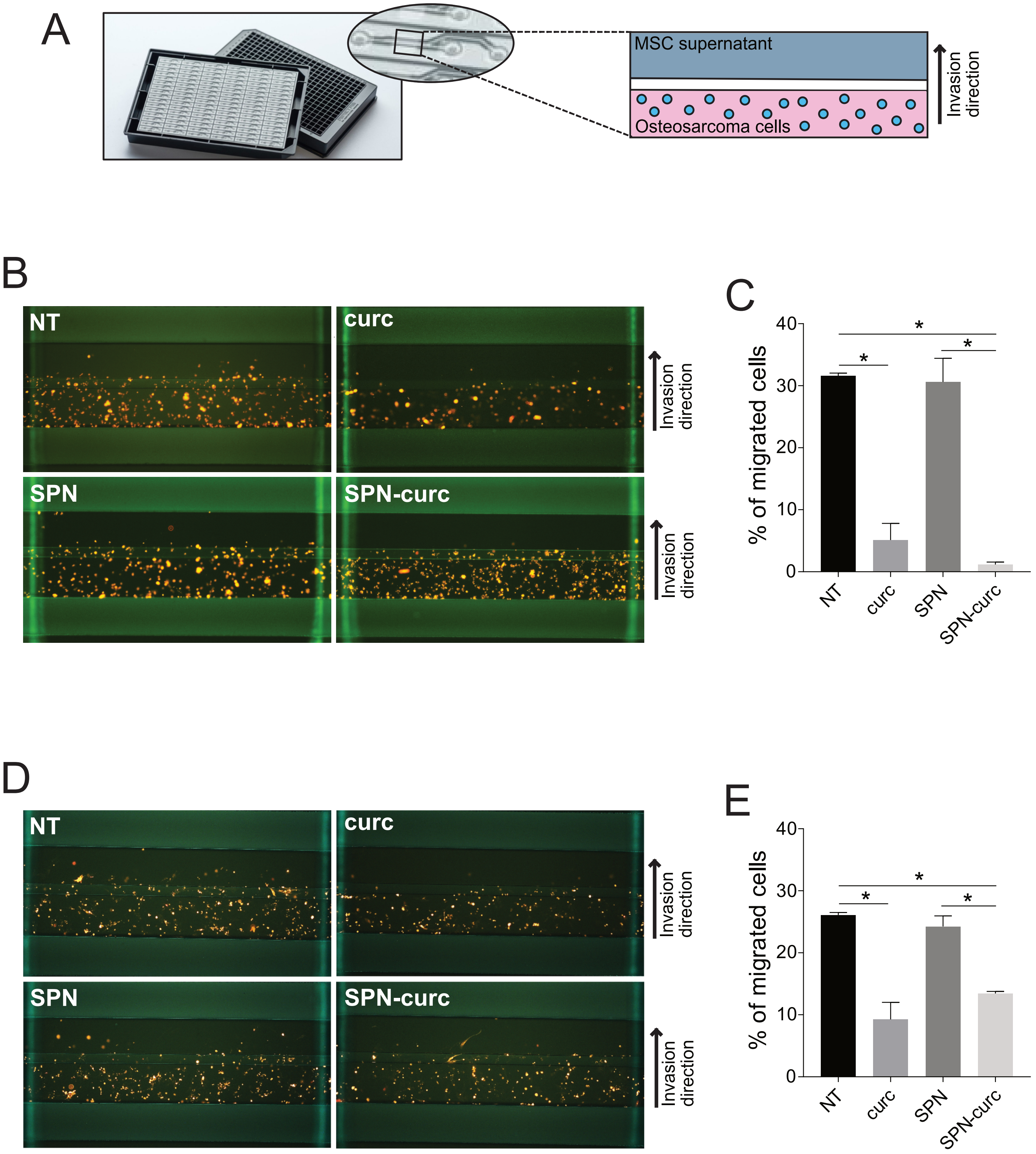

Figure S2. SPN-curc impaired the pro-tumorigenic activity of MSCs induced by acidosis. (**A**) Schematic representation of the microfluidic device that was used to evaluate cell invasion. Matrigel-embedded osteosarcoma cells were seeded in one of the two channels of the chi and exposed to the supernatant of MSCs treated or not with curcumin, SPN-curc, or empty SPN; Percentage of migrated and invaded 143b cells (**B**) and Saos-2 cells (**C**), expressed as the ratio between the number of cells that were migrated to the upper lane and the total number of cells within the microfluidic device x 100. Mean ± SE (n = 3, *P<0.05).
